# Supplementary figures and images for: Urine LOX-1 and Volatilome as Promising Tools towards the Early Detection of Renal Cancer
Source: Cancers (Basel). 2021 Aug 21;13(16):4213. doi: 10.3390/cancers13164213 (PMC8393749; doi:10.3390/cancers13164213)

IB:Anti LOX-1(Abcam, ab60178)

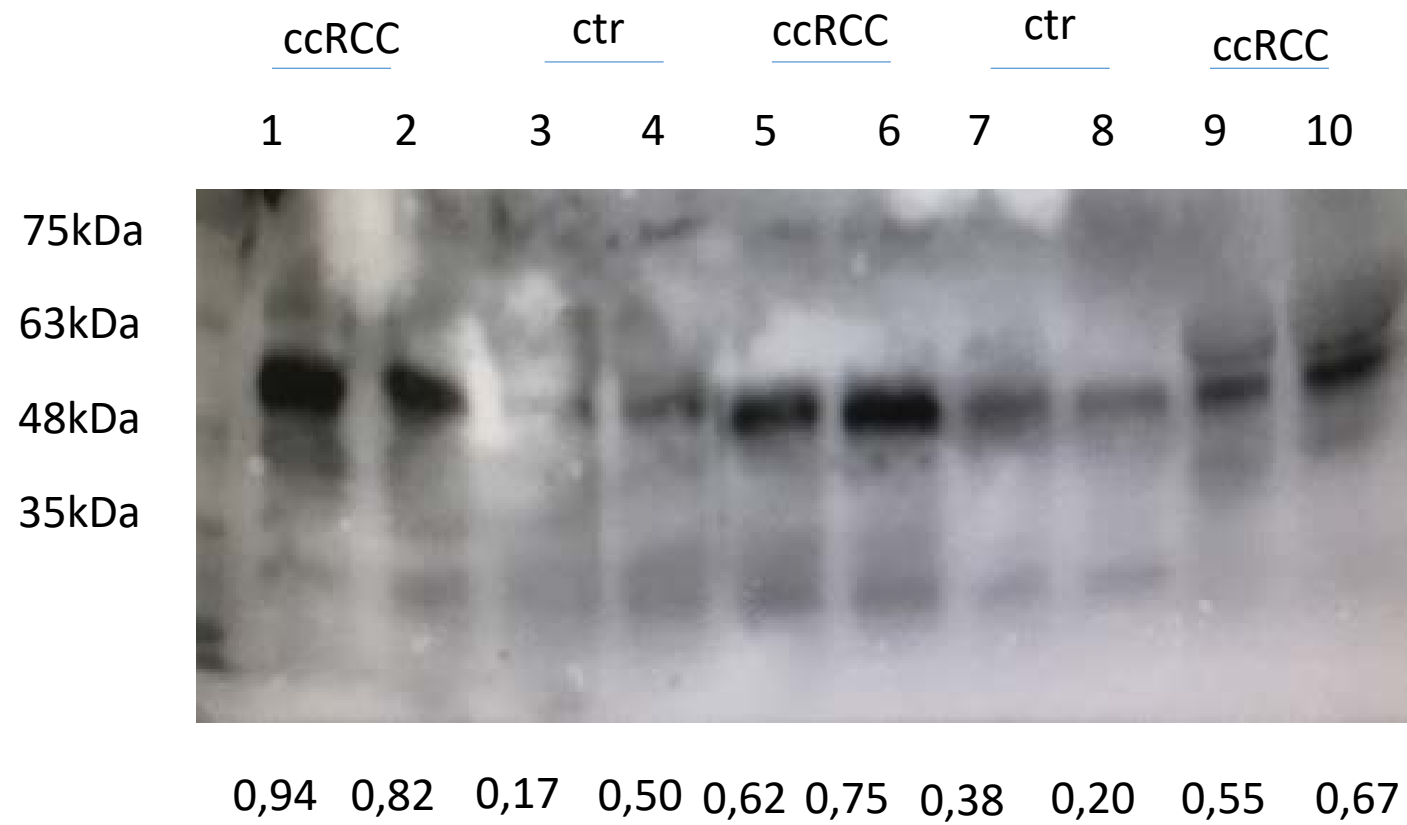

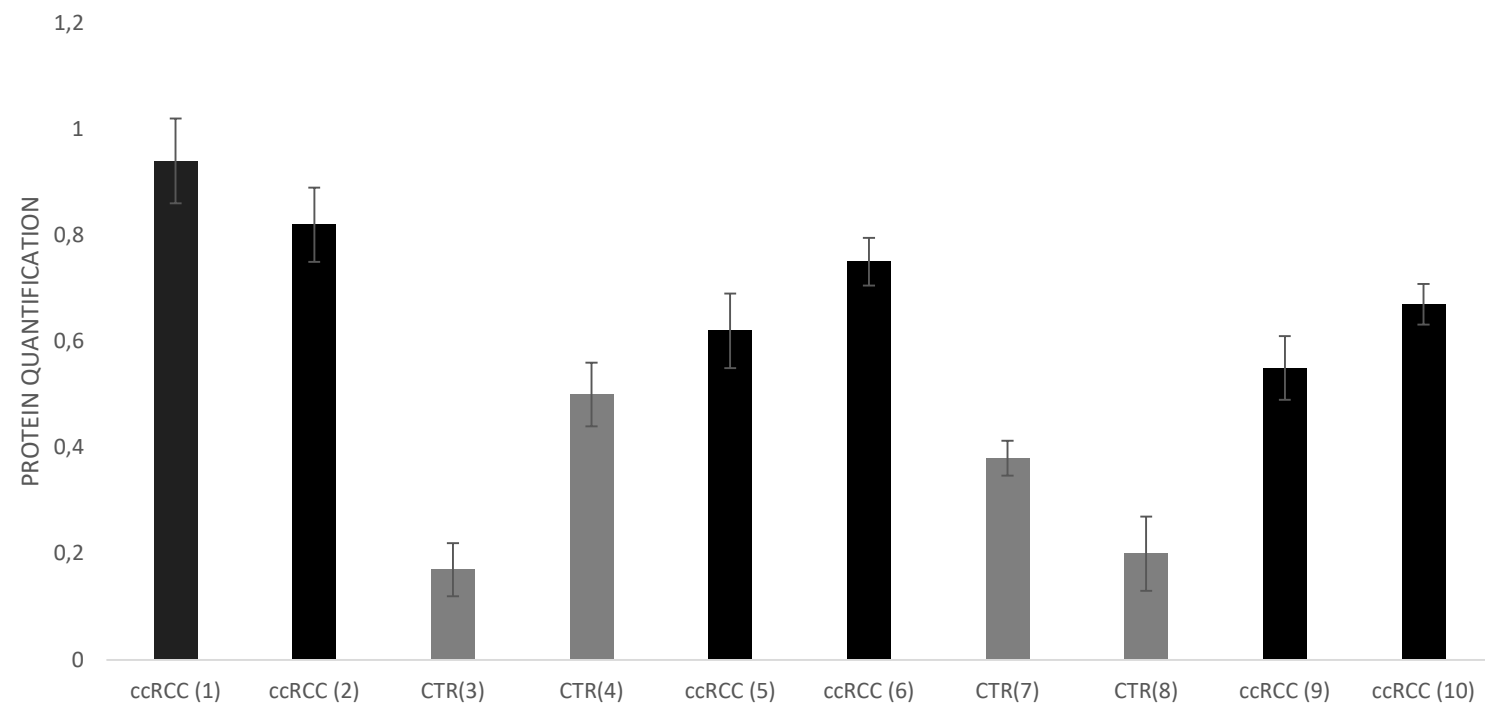

Supplement: Supplementary file 1 [file cancers-13-04213-s001.zip › cancers-1279339-supplementary.pdf]
